# Automated Voxel Placement: A Linux-based Suite of Tools for Accurate and Reliable Single Voxel Coregistration

**DOI:** 10.17756/jnpn.2018-020

**Published:** 2018-02-08

**Authors:** Eric A. Woodcock, Muzamil Arshad, Dalal Khatib, Jeffrey A. Stanley

**Affiliations:** Brain Imaging Research Division, Department of Psychiatry and Behavioral Neurosciences, Wayne State University School of Medicine, Detroit, MI 48201, USA

**Keywords:** Magnetic resonance spectroscopy, Single voxel, Automated voxel placement, Coregistration, Neurochemistry

## Abstract

**Background:**

Single-voxel proton magnetic resonance spectroscopy (^1^H MRS) is a powerful technique for studying *in vivo* neurochemistry, but has an often-overlooked source of error variance: inconsistent voxel placement between scans. We developed and evaluated an Automated Voxel Placement (AVP) procedure for accurate and reliable ^1^H MRS voxel prescription. AVP is a suite of Linux-based programs that facilitate automated template-driven single-voxel coregistration.

**Methods:**

Three studies were conducted to evaluate AVP for prescription of one voxel: left dorsolateral prefrontal cortex. First, we evaluated how robust AVP was to ‘extreme’ subject head positions/angulations within the scanner head coil. Second, subjects (N = 13) were recruited and underwent MR scans. Manual voxel prescription (n = 5) was contrasted with AVP (n = 8). A subset of AVP subjects (n = 4) completed a second scan. Third, ongoing data collection (n = 16; recruited for a separate study) helped evaluate AVP. Voxel placement accuracy was quantified as 3D geometric voxel overlap percentage between each subject’s voxel and the template voxel. Reliability was quantified as 3D geometric voxel overlap percentage across subjects at each time point and within subjects who completed two scans.

**Results:**

Results demonstrated that AVP was robust to ‘extreme’ head positions (97.5% - 97.9% overlap with the template voxel). AVP was significantly more accurate (baseline and follow-up: 96.2% ± 3.0% and 97.6% ± 1.4% overlap) than manual voxel placement (67.7% ± 22.8% overlap; *p*s<.05). AVP was reliable within- (97.9%) and between-subjects (94.2% and 97.2% overlap; baseline and follow-up; respectively). Finally, ongoing data collection indicates AVP is accurate (96.0%).

**Conclusion:**

These pilot studies demonstrated that AVP was feasible, accurate, and reliable method for automated single voxel coregistration.

## Introduction

*In vivo* proton magnetic resonance spectroscopy (^1^H MRS) is a powerful non-invasive neuroimaging technique that can quantify neurochemical levels in localized brain areas [1-3]. For example, ^1^H MRS can distinguish tumor from healthy brain tissue [4, 5], locate tissue affected by seizure disorders [6-9], and identify cerebral atrophy associated with dementia and mild cognitive impairment [10-16]. In addition, ^1^H MRS has been used to track disease progression [14, 17, 18] and treatment response over time within subjects [19, 20].

Single-voxel ^1^H MRS (SVS) facilitates measurement of biochemical information in a three-dimensional volume (voxel) located in a specific anatomical region. However, inconsistent and unreliable voxel placement between subjects in cross-sectional studies (i.e., to contrast neurological/psychiatric diagnoses or medication responses) or within subjects across time in longitudinal studies (i.e., treatment response) is a source of error variance that is often overlooked in SVS studies [21, 22]. It is well-established that neurochemistry varies by tissue type (grey matter vs. white matter vs. cerebrospinal fluid) and anatomical location [23, 24]. Indeed, prior research has demonstrated that metabolite levels differed by up to 30% depending on voxel placement; thus, highlighting the importance of accurate and reliable voxel placement for SVS [25-27]. Unfortunately, SVS studies often rely on manual voxel prescription by an operator for each research subject based on (subjective) anatomical features. This process can be time-consuming, inconsistent, and challenging. Moreover, SVS studies often do not report voxel placement reliability and ignore the error variance contributed by inconsistent voxel placement. In the few published studies that reported percentage of 3D geometric voxel overlap between subjects, overlap was ~70% [28] or worse [29].

Several techniques have been developed to address the problem of voxel placement inconsistency, including methods by Hancu et al. and Storrs et al. The Hancu method [30, 31] used a registration algorithm to automatically reposition the manually-placed voxel from the subject’s first scan. This approach was highly-reliable (94% - 95% within-subject voxel overlap). However, this approach required manual placement for each subject’s first scan: thus, limiting its utility in research settings [30, 31]. At the time of publication, the authors were able to find one automated method capable of between-subject voxel coregistration: the Storrs method [29]. The Storrs method used a template-drive and demonstrated excellent within-subject voxel replacement reliability: 97% - 98% (depending on anatomical location), but modest between-subject median voxel placement accuracy (overlap with template voxel): 81% - 84% (depending on anatomical location) [29]. Voxel overlap shared across subjects (i.e., between-subject reliability) was not reported for the Storrs method. Inconsistent and unreliable voxel placement between subjects remains a significant obstacle for SVS research studies.

The aim of the present study was to develop and evaluate an automated voxel placement technique for reliable voxel coregistration within- and between-subjects. Automated Voxel Placement (AVP) is a suite of Linux-based computer programs/scripts designed to promote implementation using existing coregistration algorithms. An a *priori* voxel location in the left dorsolateral prefrontal cortex (dlPFC) was selected (challenging location for manual voxel placement) and evaluated. Two studies evaluated the AVP method. First, we conducted a ‘proof-of-principle’ study to evaluate how robust AVP coregistration was to ‘extreme’ subject head positions within the head coil. Second, a sample of research subjects (N = 13) were recruited to evaluate the efficacy of the AVP approach (n = 8) vs. manual placement (n = 5). We hypothesized that the AVP approach would be significantly more accurate and reliable than the most commonly used approach: manual voxel placement.

## Materials and Methods

### Participants

All procedures performed in studies involving human participants were in accordance with the ethical standards of the institutional and/or national research committee and with the 1964 Helsinki declaration and its later amendments or comparable ethical standards. Thirteen volunteers were recruited locally to participate in this research study and were compensated for their time. Informed consent was obtained from all individual participants included in the study. All 13 subjects completed the baseline MRI scan. Manual voxel prescription was used for five subjects, while AVP was used for the remaining eight subjects. In addition, a subset of AVP participants (n = 4) completed a follow-up MRI scan ~2.5 days later (on average) to evaluate AVP’s within-subject reliability.

An experienced MR technologist (DK) used a 2D printout that depicted the optimal voxel location in template brain space along three orthonormal directions (sagittal, coronal, and axial). The voxel was prescribed using a wireframe depiction of the voxel location on a computer system display along three orthonormal directions. The Principal Investigator (EW) confirmed each voxel placement before it was finalized. This procedure was replicated for all five subjects who received manual voxel prescription.

### AVP suite

The AVP suite is available free-of-charge: https://github.com/ewoodcock/avp_scripts.git. AVP was developed and evaluated using an adult template brain (‘LAS’ image orientation) on a 3 Tesla Siemens Verio system. However, the AVP method was designed to be scanner system-, field strength-, subject population-, and anatomy-independent. Three programs are included in the AVP suite: ‘AVP_Create’, ‘AVP_Coregister’, and ‘AVP_Overlap.’

### AVP_Create

**Figure 1:**
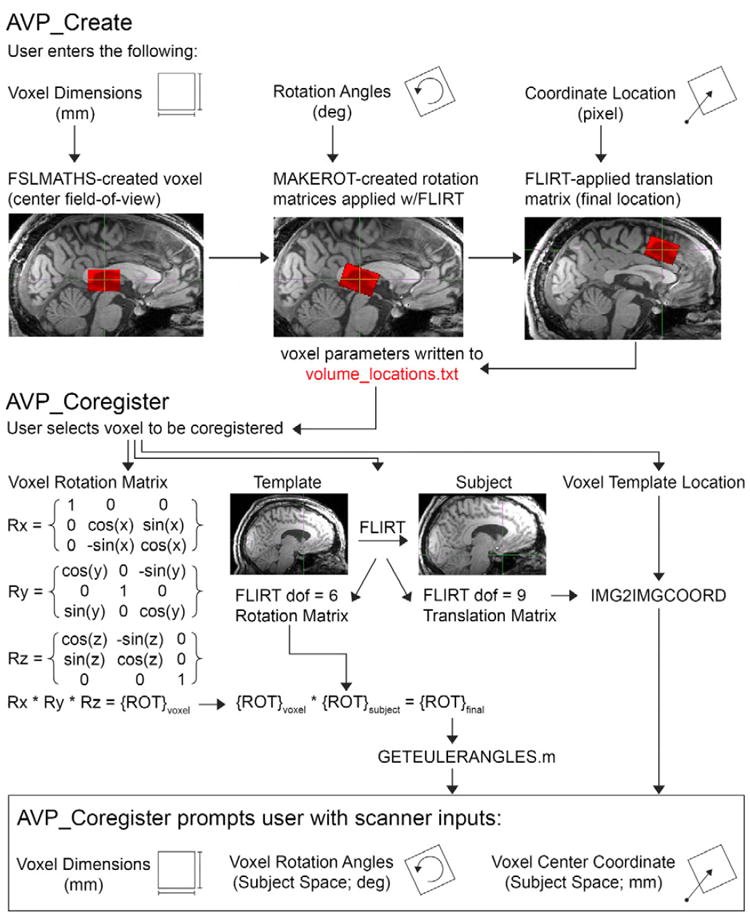
**Upper panel:** A schematic diagram of the ‘AVP_Create’ program is depicted. The user enters voxel dimension, rotation angles (about each axis), and voxel center coordinate. ‘AVP_Create’ creates the voxel in the center of the FOV. The voxel is rotated in the center of the FOV and then translated to its final location. **Lower panel:** A schematic diagram of the ‘AVP_Coregister’ program is depicted. The user selects a template voxel for coregistration to the present research subject. ‘AVP_Coregister’ will calculate the center voxel location (‘FLIRT’ dof = 9 and ‘img2imgcoord’) and rotation angles (‘FLIRT’ dof = 6) in subject space. ‘AVP_Coregister’ will prompt the user with the scanner values (voxel dimensions, rotation angles, and voxel center coordinate) needed to prescribe the voxel in subject space.

A schematic diagram of the processing logic used in ‘AVP_Create’ is depicted in figure 1. Upon execution of the ‘AVP_Create’ script, the user will be prompted to enter voxel parameters, including: voxel location (center coordinate), voxel dimensions, voxel angulation/rotation about each axis [T>S (x), T>C (y), Rotation (z)], voxel description and study name, and image orientation (e.g. ‘LAS’). ‘AVP_Create’ will generate the specified volume and prompt the user to visually appraise it in the template image space using the 3D viewer (FSLView; FMRIB, Oxford, UK). If the voxel position is inadequate, the user can make the necessary adjustments. This is an iterative process and will likely require several adjustments (<1 min computer processing time per adjustment). Once the voxel position is optimal, all voxel information is stored in the ‘voxel_locations.txt’ file for future coregistration. Additional voxel locations can be created using ‘AVP_Create’ by repeating this process. In this way, the user can generate a library of study-specific template voxels (each with a study-specific template image) that are retained for future coregistration.

### AVP_Coregister

Upon execution of the ‘AVP_Coregister’ script, the user must input the study name, subject ID, and time point for the current research subject (Figure 1), and select the template voxel for coregistration. The template anatomical image was coregistered (rigid; 6 degrees of freedom [dof]; ‘FLIRT’ [32]) to the subject T_1_-weighted anatomical image. The default cost function (adopted herein) used by ‘FLIRT’ is Correlation Ratio with trilinear interpolation. ‘FLIRT’ combines a local and global search strategy to optimize coregistration. The rigid-body coregistration matrix (linear translation and rotation adjustments along all three axes) was inverted and used to calculate Euler rotation angles. A second linear (affine) coregistration (dof = 9; ‘FLIRT’) and ‘img2imgcoord’ function were used to calculate voxel center coordinates in subject space. Nine dof limited ‘FLIRT’ to linear translation, rotation, and scaling adjustments along all three axes to coregister the images. Finally, those parameters (voxel dimensions, center coordinate, and rotation angles) were input at the scanner. The authors recommend visual appraisal to confirm the voxel placement is optimal.

### AVP_Overlap

**Figure 2:**
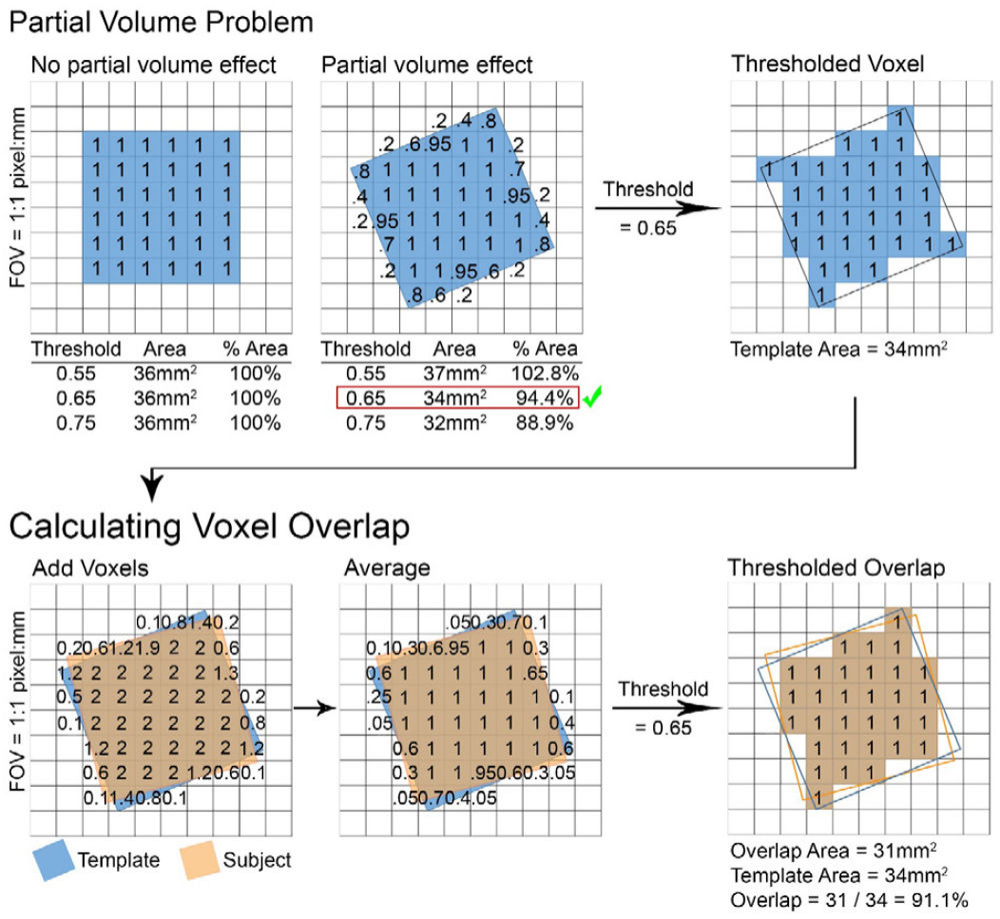
**Upper panel:** A schematic representation of the ‘partial volume problem’ is depicted. On the left, a blue 6 mm × 6 mm 2D voxel is orthogonal to the Field-of-View (FOV; i.e., unrotated). The voxel occupies 100% of the 2D pixel space in every pixel location, and thus, every location has a pixel intensity value = 1 (area = 36 mm^2^) at all pixel intensity threshold levels (i.e., no partial volume effect). In the upper middle panel, the 2D voxel is rotated with respect to the FOV, thus creating a ‘partial volume effect.’ Along the edges of the voxel, pixel intensity values range between 0 and 1. Thus, depending on the pixel intensity threshold (i.e. the threshold beyond which the voxel is deemed present [vs. not present] in each pixel location), the voxel area will range between 37 mm^2^ (102.8% of ‘true’ template voxel area; i.e., its area when orthogonal to the FOV or 36 mm^2^) and 32 mm^2^ (88.9%). The user should select the pixel intensity threshold that corresponds to closest to, without exceeding, 100% of the unrotated area (i.e., 36 mm^2^). The appropriate pixel threshold is 0.65 which corresponds to a thresholded voxel area of 34 mm^2^ (figure on the upper right) or 94.4% of the template area (i.e., 34 mm^2^/36 mm^2^). The user should report voxel overlap results that correspond to the selected pixel intensity threshold. **Lower panel:** The analytic strategy for calculating voxel overlap with the template voxel (i.e., voxel placement ‘accuracy’) is depicted. The binarized subject voxel is added to the binarized template voxel in template space (after coregistration using ‘AVP_Overlap’) such that pixel values sum. At every pixel location, an average pixel value is calculated and thresholded (at the selected pixel intensity threshold: 0.65). The overlap area is quantified and expressed as a percentage of the template area (using the same pixel intensity threshold: 0.65). In this example, the thresholded overlap area is 31 mm^2^, which is divided by the template area (34 mm^2^), and expressed as a percentage: 91.1% voxel overlap. This example depicts calculation of voxel placement accuracy: subject voxel overlap percentage with the template voxel. However, the same steps are repeated for between-subject overlap and within-subject overlap, with one difference – only subject voxels are used for those metrics. The template voxel is not included in the calculation of between- or within-subject overlap, only for calculation of voxel placement ‘accuracy’.

Upon execution of ‘AVP_Overlap’, voxel attributes were extracted from the dicom file header information for each subject at each time point. Thus, voxel overlap was calculated based on the region from which MRS spectra were acquired in each subject, and not values or transformation matrices calculated by ‘AVP_Create’ or ‘AVP_Coregister’. The processing pipeline for ‘AVP_Overlap’ mirrored ‘AVP_Coregister’ and used two coregistration procedures (rigid-body dof = 6 ‘FLIRT’ for calculation of Euler angles [rotation matrix] and linear dof = 9 ‘FLIRT’ for calculation of voxel coordinate location [translation matrix]). Next, the voxel was ‘reconstructed’ in template space using the prescribed voxel dimensions. This facilitated accurate calculation of rotation angles and voxel center coordinate that is calibrated for anatomical size differences (between the template and subject anatomy) while preserving voxel dimensions. Finally, the voxel is binarized: every pixel location has a value between 0 (voxel not present) and 1 (voxel present). It was important to consider the ‘partial volume’ problem. Any voxel that is defined as a binary mask (ones and zeroes) and is rotated/angulated with respect to the Field-of-View (FOV) will have pixels along the edges with pixel intensity values somewhere between 0 and 1 due to the resampling of the voxel mask (Figure 2; upper panel). To minimize the impact of this effect on the accuracy of voxel overlap calculation, two approaches were implemented. First, the entire matrix was resampled at a pixel resolution of 0.5 mm isotropic to minimize partial volume effects. Second, a range of pixel intensity thresholds are calculated in the ‘AVP_Overlap’ script. The user should select the pixel intensity threshold closest to, without exceeding, 100% of the unrotated template voxel size (labeled ‘Percent Total Voxel’ in the ‘Overlap_Summary. txt’ file) for all voxel overlap metrics. If the template voxel is not rotated (i.e., orthogonal to the FOV), the user should select the highest pixel intensity threshold provided (0.95).

‘AVP_Overlap’ automatically calculates several metrics for evaluation of voxel placement. 1) Voxel Placement ‘Accuracy’. This is defined as the percentage of 3D geometric voxel overlap between a subject’s voxel and the template voxel. The binarized subject voxel is added to the binarized template voxel in template space such that pixel values sum. Next, the average pixel value in every pixel location is calculated (Figure 2; lower panel). The number of pixels from the averaged image that exceed the pixel intensity threshold are quantified and divided by the number of pixels in the template voxel (in isolation) that exceed the pixel intensity threshold. This process is repeated separately for every subject voxel before a group mean is calculated and expressed as a percentage. If more than one experimental time point exists (i.e., longitudinal study), this process is repeated separately for each time point. 2) Between-Subject Overlap. Every binarized subject voxel is summed in template space and an average pixel value is quantified for every pixel location. Again, the number of pixels that exceed the pixel intensity threshold from the averaged image is divided by the number of pixels in the template voxel (in isolation) that exceed that threshold and is expressed as a percentage. This process is repeated separately for each experimental time point. 3) Within-Subject Overlap. If multiple experimental time points exist, voxel overlap across time points is calculated separately for each subject. Binarized subject voxels from each time point are summed, pixel values averaged, and then thresholded (again, using the pixel intensity threshold). Next, summary statistics are calculated across subjects: group mean % within-subject voxel overlap and coefficient of variation (CV%). In addition, partial volume tissue segmentation maps (using ‘FAST’; estimated from T_1_-weighted scan) within the voxel space are calculated for each subject. Group mean percentage of gray and white matter and cerebrospinal fluid are reported for each experimental time point.

The authors note that RF pulses are imperfect (especially 180° RF pulses; i.e., PRESS), and thus, are associated with rounded MRS voxel edges. However, for purposes of geometric voxel overlap calculation, all voxels were assumed to be perfectly rectangular with 90° angles and flat edges.

### AVP computer processing

‘AVP_Create’ and ‘AVP_Coregister’ were executed on a laptop computer with an Intel Core i7 2.60 GHz processer and 64-bit OS running Windows version 8.1 (Microsoft Corporation, Redmond, WA). The scripts were executed in an Oracle Linux Virtual Machine (Oracle Corporation, Redwood Shores, CA; 4 GB of dedicated RAM). Computer processing time for ‘AVP_Create’ and ‘AVP_Coregister’ were acceptable (<1 minute and ~2 minutes, respectively).

### Neuroimaging

All imaging was conducted on a 3 Tesla Siemens Verio system with a 32-channel receive-only volume head coil. High resolution T_1_-weighted structural scans were collected using the 3D Magnetization Prepared Rapid Gradient Echo (MPRAGE) sequence with the following parameters: TR = 2.2 s, TE = 3 ms, T_I_ = 799 ms, flip angle = 13°, FOV = 256 × 256 × 160 mm, 256 × 1 mm thick axial slices, matrix = 176 × 256.

### Voxel placement

**Figure 3:**
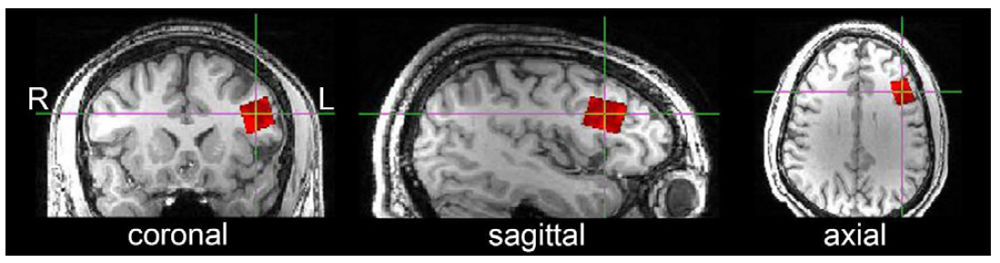
The voxel investigated in this study is depicted in orthonormal slices. The voxel is located primarily in Brodmann Areas 45 and 46 (dlPFC; 1.5 × 2.0 × 1.5 cm).

The voxel location evaluated in this study is depicted in figure 3. Voxel dimensions were 1.5 × 2.0 × 1.5 cm^3^ (volume = 4.5 cm^3^). The voxel was located in the left dlPFC (Brodmann Areas 45 and 46). The dlPFC was selected because it is a challenging location for consistent manual voxel placement.

### Proof-of-principle

**Figure 4:**
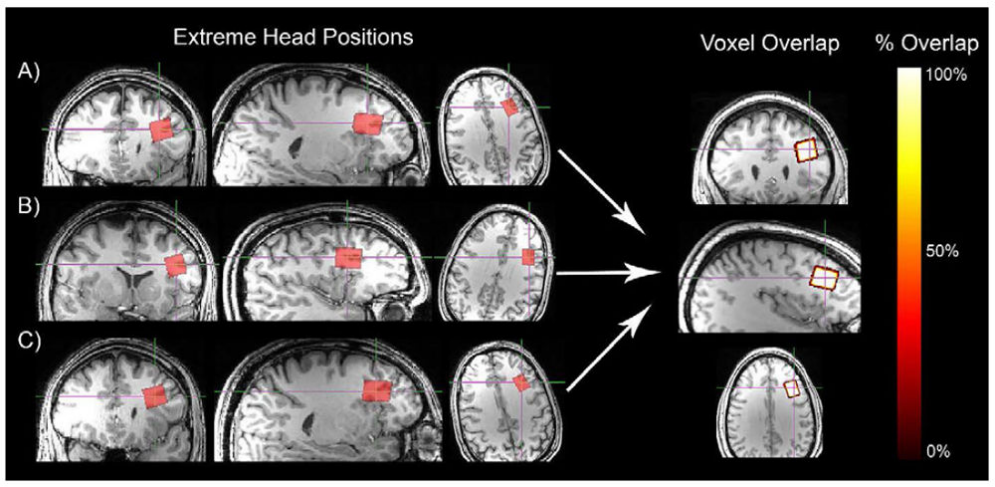
**Left panel:** Orthonormal slices of the three extreme head positions evaluated during the ‘proof-of-principle’ investigation are depicted. **Right panel:** Using voxel parameter information from the dicom file generated during ^1^H MRS acquisition, each voxel was recreated in subject space, coregistered to template space, and 3D geometric voxel overlap was evaluated. **Note:** white indicates complete voxel overlap, yellow-orange gradient indicates incomplete voxel overlap, and red indicates no voxel overlap.

As a ‘proof-of-principle’ evaluation, we evaluated three ‘extreme’ head positions for a single subject (Figure 4) to evaluate how robust AVP was to subject head position in the head coil. The following protocol was repeated three times during a single scanning session (~45 min): 1) the technologist positioned the subject in the scanner, 2) T_1_-weighted structural images were collected (using MPRAGE), and 3) the template voxel (Figure 3; left dlPFC) was coregistered and prescribed (using ‘AVP_Coregister’). Three different analyses were conducted to evaluate the reliability of ‘AVP_Coregister’ for this ‘proof-of-principle’ investigation. First, percentage of 3D geometric voxel overlap between each of the three voxel placements and the template voxel was calculated (i.e. ‘accuracy’). Second, percentage of 3D geometric overlap and CV% across the three voxels was calculated (i.e. ‘reliability’). Third, voxel tissue composition was calculated and evaluated for consistency (CV%).

### Ongoing data collection

In subsequent research studies conducted in our laboratory, the identical left dlPFC voxel was prescribed using AVP. Thus, to evaluate the reliability of AVP in a larger sample, we included an additional 16 subjects (24 subjects in total) and evaluated between-subject voxel overlap.

### Analysis strategy

Results were calculated using the ‘AVP_Overlap’ script. The primary outcome variables for this study were: 1) voxel placement ‘accuracy’, 2) between-subject voxel overlap (i.e., ‘reliability’), and 3) within-subject voxel overlap, each of which was defined above (see ‘AVP_Overlap’). For the present voxel, a 0.65 pixel threshold was selected (97.2%-98.7% of the unrotated template voxel size). We report mean percentage of voxel placement accuracy and between-subject voxel overlap separately for each timepoint: baseline and followup. For within-subject overlap, we report mean percentage of within-subject overlap and CV% (for subjects who completed both scans). In addition, using the partial volume tissue segmentation maps within the voxel space, we calculated CV% between baseline and follow-up scans for gray and white matter (i.e., ‘within-subject anatomical consistency’).

Results using manual voxel placement (n = 5) were contrasted with the AVP approach at baseline (n = 8) and follow-up (n = 4) using one-way analyses of variance. Descriptive statistics are presented as mean (M) ± one standard deviation (SD; unless otherwise noted).

## Results

### Proof-of-principle

Prior to subject recruitment or enrollment, AVP was evaluated on a single subject during a single scanning session using three different ‘extreme’ head positions in the head coil (Figure 4). Table 1 describes the translational and rotational differences for each of the three head positions relative to the template voxel. 3D geometric voxel overlap between each head position and the template voxel demonstrated that AVP was highly accurate (median: 97.7%; range: 97.5%-97.9% overlap). Mean 3D geometric voxel overlap across all three head positions demonstrated AVP was highly reliable (98.9% overlap) and consistent (CV% = 0.19%). Tissue segmentation results demonstrate that anatomical composition across the three head positions was consistent (grey matter CV% = 1.63%, range = 24.8%-25.8%; white matter CV% = 0.84%, range = 72.9%-74.4%). Together, these data demonstrated that the AVP approach was robust to subject head position in the head coil.

### Participant characteristics

Thirteen subjects were recruited for formal evaluation of AVP. The modal participant was a 28-year-old (mean ± 1 SD: 27.7 ± 3.8 yrs old; range: 21-34 yrs) African-American (76.9%) male (84.6%).

### Voxel placement accuracy

**Figure 5:**
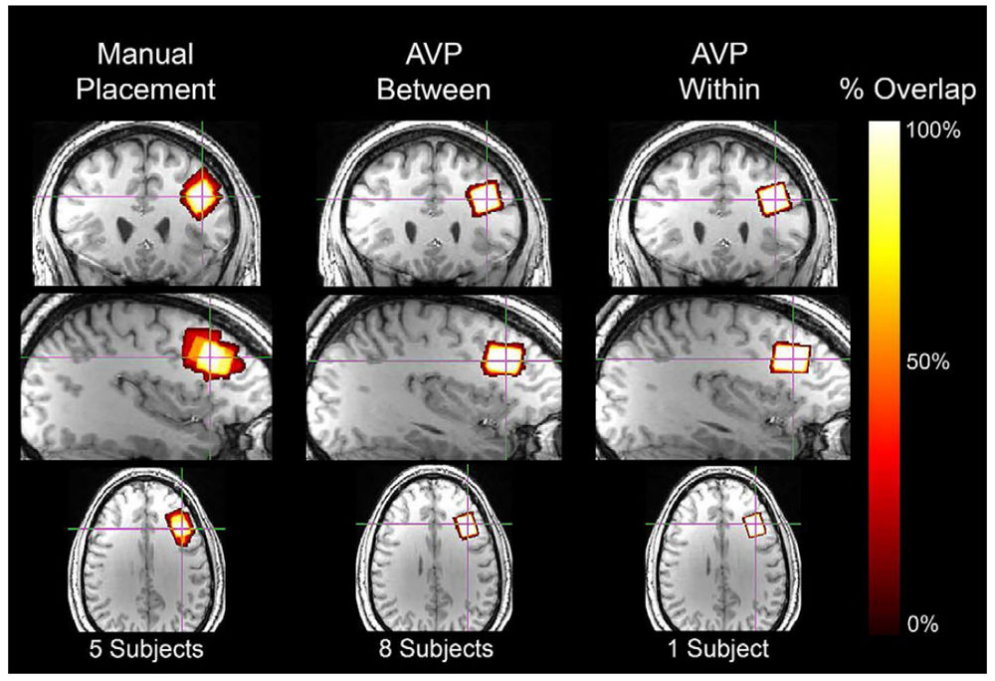
Orthonormal slices of 3D geometric voxel overlap in template space are depicted. Left panel: Voxel overlap across subjects using manual voxel placement (n = 5) is depicted. Center panel: Voxel overlap across subjects using AVP (n = 8; baseline) is depicted. Right panel: Voxel overlap within a single subject across timepoints using AVP is depicted. Note: white indicates complete voxel overlap, yellow-orange gradient indicates incomplete voxel overlap, and red indicates no voxel overlap.

AVP was more accurate than manual voxel placement (n = 5) at baseline (n = 8) and follow-up (n = 4): *F* (1, 12) = 12.84, *p* < .005 and *F* (1,8) = 6.76, *p* < .05, respectively (Figure 5). Mean accuracy (± 1 SD) using AVP was higher (96.2% ± 3.0% and 97.6% ± 1.4%; baseline and follow-up, respectively) than manual voxel placement (67.7% ± 22.8%).

### Within-subject reliability

A subset of participants in the AVP condition returned for a follow-up visit (n = 4). Mean within-subject overlap using AVP was 97.3% ± 2.1%. Mean gray and white matter percentage of voxel composition were: 32.2% (range: 20.4%- 38.3%; CV% = 10.8%) and 65.5% (range: 58.5%-79.5%; CV% = 5.2%), respectively.

One outlier for gray matter (CV% = 28.3%; all other values <10%) was observed. This value was associated with relatively poor within-subject reliability (93.8% overlap). In this subject, percentage of voxel composed of gray matter was estimated to be 36.5% at baseline and 20.4% at follow-up. This outlier illustrated that small voxel coregistration errors can influence voxel tissue composition, which, in turn, will bias estimates of neurochemistry.

### Between-subject reliability

Manual voxel placement resulted in poor between-subject reliability: 67.7%. However, this value was consistent with the literature for manual voxel placement (~70% between-subject voxel overlap) [28, 33, 34]. Mean between-subject overlap using AVP was 94.2% at baseline and 97.2% at follow-up.

### Ongoing data collection

**Figure 6:**
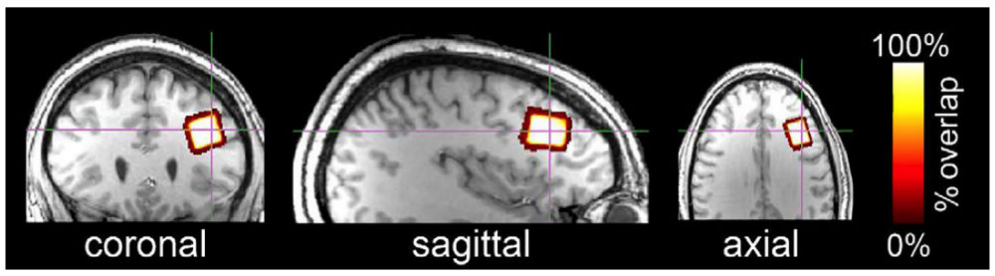
Orthonormal slices of 3D geometric voxel overlap (n = 24) in template space are depicted.

To date, AVP has been used to prescribe this identical left dlPFC voxel in 24 subjects in subsequent research studies in our laboratory. Results indicated 3D geometric voxel overlap percentage (between-subject reliability) was excellent across 24 subjects (96.0%; Figure 6).

## Discussion

In this study, we evaluated a method for automated voxel placement using a prospective, template-driven approach. The AVP suite facilitated creation of template voxel positions that could be automatically coregistered and prescribed at the scanner (~2 min computer processing time). Results indicate AVP resulted in highly accurate and reliable voxel coregistration. AVP used existing coregistration algorithms in a novel and freely-available package designed to promote implementation, especially in clinical SVS research studies.

Results from this study demonstrated that AVP was feasible and accurate. ‘AVP_Create’ facilitated creation of a library of template voxels that were retained for future coregistration. A different template image can be designated for each research study, subject population, and anatomical region of interest. Prior to subject scanning, users appraise and iteratively adjust each template voxel location (using a 3D viewer) until optimal. This process saved valuable scan time and facilitated accurate template voxel placement (less error prone than manual voxel placement guided by 2D anatomical images). At the scanner, ‘AVP_Coregister’ facilitated accurate voxel prescription based on the subject’s T_1_-weighted anatomical scan. A rigid-body (dof = 6) coregistration procedure was used to calculate Euler rotation angles, while a linear procedure (dof = 9) was used to calculate voxel center coordinates in subject space. This approach provided an optimal balance between efficiency (computer processing time = ~2 minutes) and accuracy. Mean 3D geometric voxel overlap between each subject voxel and the template voxel (i.e. voxel placement ‘accuracy’) was 96.2% at baseline and 97.6% at follow-up. Importantly, overlap was calculated based on the actual voxel location from which spectra were acquired (parameters extracted from each subject’s dicom header file) and not matrices or coordinates generated by AVP. These data demonstrated that the median subject voxel placement was inaccurate approximately 0.15 mm in a given direction relative to the template voxel (likely due to rounding and partial volume effects). At this level of accuracy, it is likely that subject motion will contribute greater voxel placement error. In contrast, manual voxel placement yielded mean overlap accuracy of 67.7% (~2 mm placement error in a given direction; comparable to prior research [28, 33]). Finally, AVP was substantially more accurate voxel coregistration than the most accurate published approach known to the authors (~13% overlap improvement; Cohen’s d ≥ 2.3 [large effect]) [29].

In addition to placement accuracy, we calculated within-subject voxel overlap reliability and voxel tissue composition consistency across the two scans (~2.5 days apart). Within-subject AVP coregistration across subjects was highly reliable and consistent (mean: 97.3%). These data indicated that mean voxel replacement inaccuracy was approximately 0.2 mm in any given direction. AVP was roughly equivalent to the most reliable published approach [29]. Voxel tissue composition (percentage of grey vs. white matter) was consistent within individuals across scanning sessions and during the ‘extreme’ head position ‘proof-of-principle’ test.

Finally, we calculated between-subject voxel overlap (mean voxel space shared across subjects in template space). Our highly-experienced SVS research team demonstrated poor between-subject reliability using manual voxel prescription guided by 2D anatomical images (overlap: 67.7%). In contrast, AVP was highly reliable and consistent at each time point (mean overlap: 94.2% and 97.2%; baseline and follow-up, respectively). Between-subject reliability was not reported for the Storrs method [29].

There exist other sophisticated automated coregistration approaches in the literature. Van der Kouwe et al. developed an automated real-time (while the subject is in the scanner) procedure in which they collect two medium resolution, large FOV images (two different tissue contrasts) and use a rigid-body transformation matrix (similar to the procedure described herein) to coregister each subject to a pre-determined template brain [35]. This procedure offers similar advantages (real-time coregistration to a template brain) as our approach. It is difficult to compare this approach with the AVP method because the authors did not evaluate voxel overlap. However, we believe the AVP method facilitated superior voxel placement reliability. Van der Kouwe and colleagues found that within-subject whole-brain test-retest reliability was significantly improved by a post-scan ‘FLIRT,’ which indicated the presence of error for within-subject coregistration [35]. AVP does not benefit from a post-scan ‘FLIRT’ for either within-subject or between-subject voxel replacement.

The authors were aware of other automated voxel placement methods [34, 36-40]. These approaches were not discussed in this paper for two reasons: 1) similar to others described in this manuscript, and/or 2) notable limitations existed relative to approaches described herein (e.g. more time-consuming, less reliable, etc.). This study had several limitations. This study included a small number of research subjects (though subsequent data collection indicated high reliability across 24 subjects) and only one voxel location. However, AVP demonstrated robust and statistically-significant superiority to manual voxel placement. AVP has not been tested on subjects with significant morphological changes (e.g., brain atrophy, tumor, or injury), but the authors presume accuracy/reliability would be impaired due to coregistration imperfections between the subject’s brain and the template.

## Conclusion

In summary, single voxel MRS research studies suffer from an often-overlooked source of error variance: inconsistent voxel placement. To combat this problem, we developed an approach that leveraged existing coregistration algorithms in a user-friendly package to facilitate automated template-driven voxel prescription and post-scan voxel overlap calculation. Our results demonstrated that AVP was significantly more accurate and reliable than the most commonly-used voxel placement method (manual prescription guided by anatomical images) and the most accurate published method (Storrs [29]) known to the authors. AVP can be easily implemented across scanner platforms (though not currently compatible with General Electric systems), field-strength, subject population, anatomy, and is available free-of-charge: https://github.com/ewoodcock/avp_scripts.git.

## Figures and Tables

**Table 1:** Extreme head positions.

	Voxel Center Coordinate (mm)			Rotation Angles (deg)		
	X (L-R)	Y (A-P)	Z (F-H)	T>S	T>C	Rotation
Template	32	25	22.5	7.0	20.0	15.0
Extreme A	23	20	35	16.9	5.8	25.3
Extreme B	50	-1.4	27	15.9	3.3	-1.7
Extreme C	19	13	32	15.8	1.7	27.4
	Δ Translation (mm)			Δ Rotation (deg)		
Template to A	-9	-5	12.5	9.9	-14.2	10.3
Template to B	18	-26.4	4.5	8.9	-16.7	-16.7
Template to C	-13	-12	9.5	8.8	-18.3	12.4

**Note:** The voxel center coordinate and rotation angles for each of the ‘extreme’ head position scans in subject space are depicted in the upper rows. The translational and rotational differences for each voxel relative to the template are depicted in the lower rows.
